# Supplementary material for: Which breathlessness dimensions associate most strongly with fatigue?–The population-based VASCOL study of elderly men
Source: PLoS One. 2023 Dec 20;18(12):e0296016. doi: 10.1371/journal.pone.0296016 (PMC10732411; doi:10.1371/journal.pone.0296016)
Supplement: Attachment — Submitted filename: Reviewers comments PLOS ONE.docx [file pone.0296016.s001.docx]

Research to improve understanding and awareness of the inter-relationship between breathlessness and fatigue is important. This has been highlighted by the Long Covid population where the 2 symptoms often co-exist and potentiate each other. However, although this study fulfils criteria for publication, I am not sure what it adds to the scientific and medical community. The main question relates to whether particular dimensions of subjective breathlessness questionnaires predict or are associated with fatigue. The limitations as I see are:

1) Why is it helpful to use a breathlessness questionnaire to understand fatigue? Would not asking about fatigue or using the FACTIT-F (or similar) be a better/more straightforward method?
2) Why chose a healthy cohort? Would it not make more sense to select a breathless group and study breathlessness questionnaires to see how they relate in this preselected population? Although 677 participants took part, only 79 had fatigue as a symptom so number relatively small in which to draw conclusions
3) The authors have stated that those with fatigue are more breathless – this seems intuitive given the increased effort put into work of breathing
4) The authors state that assessment and treatment of patients may be influenced by determining whether different dimensions of breathlessness are associated with fatigue. No example of such has been given and I cannot think of one myself. All clinical assessments of breathless patients should consider confounding factors that can increase perception of breathlessness – including pain, mood, co-morbidities and fatigue. I do not think delineating areas on a breathlessness questionnaire is the way to do this.
