# Supplementary material for: Which breathlessness dimensions associate most strongly with fatigue?–The population-based VASCOL study of elderly men
Source: PLoS One. 2023 Dec 20;18(12):e0296016. doi: 10.1371/journal.pone.0296016 (PMC10732411; doi:10.1371/journal.pone.0296016)
Supplement: Attachment — Submitted filename: Answers to editor PLOS ONE.docx [file pone.0296016.s003.docx]

# Answers to editor

1. **Please review your reference list to ensure that it is complete and correct. If you have cited papers that have been retracted, please include the rationale for doing so in the manuscript text, or remove these references and replace them with relevant current references. Any changes to the reference list should be mentioned in the rebuttal letter that accompanies your revised manuscript. If you need to cite a retracted article, indicate the article’s retracted status in the References list and also include a citation and full reference for the retraction notice.**

The reference list has been reviewed and all the references are correct - none of the references have been retracted.

2. **In the previous review I pointed out some inconsistency in numbers in table1. Authors responded saying it was updated to show the correct numbers. However I can still see some inconsistency. For example, cardiovascular disease total is not the same as the sum of those with fatigue and non-fatigue.**

We have gone through the numbers in table1 and have updated it for the correct numbers.

3. **In the previous review I queried on the Figure3 caption that refers to identifying clinically relevant fatigue level of Facit-F ≤30 using beta coefficients when both X and Y data are transformed twice before using in OLS regression. Authors responded adding a sentence to the statistical analysis section saying only the X data were transformed, Y data were not transformed. However I can still see the previous statement in the same paragraph that says both breathlessness and fatigue were transferred. Also, figure3 caption also says all scores were analysed as Z scores. the same. Authors need to proof read the manuscript to avoid contradictory statements.**

This has been addressed in the statistical analysis section and in the figure 3 captions – we greatly appreciate and thank the editor for all the comments.
